# A secure future? Human urban and agricultural land use benefits a flightless island-endemic rail despite climate change

**DOI:** 10.1098/rsos.230386

**Published:** 2023-06-28

**Authors:** Lucile Lévêque, Rahil J. Amin, Jessie Buettel, Scott Carver, Barry Brook

**Affiliations:** University of Tasmania, Hobart, Tasmania, Australia

**Keywords:** species distribution model, flightless, climate change, urban, island, bird

## Abstract

Identifying environmental characteristics that limit species' distributions is important for contemporary conservation and inferring responses to future environmental change. The Tasmanian native hen is an island endemic flightless rail and a survivor of a prehistoric extirpation event. Little is known about the regional-scale environmental characteristics influencing the distribution of native hens, or how their future distribution might be impacted by environmental shifts (e.g. climate change). Using a combination of local fieldwork and species distribution modelling, we assess environmental factors shaping the contemporary distribution of the native hen, and project future distribution changes under predicted climate change. We find 37% of Tasmania is currently suitable for the native hens, owing to low summer precipitation, low elevation, human-modified vegetation and urban areas. Moreover, in unsuitable regions, urban areas can create ‘oases’ of habitat, able to support populations with high breeding activity by providing resources and buffering against environmental constraints. Under climate change predictions, native hens were predicted to lose only 5% of their occupied range by 2055. We conclude that the species is resilient to climate change and benefits overall from anthropogenic landscape modifications. As such, this constitutes a rare example of a flightless rail to have adapted to human activity.

## 1. Introduction

Climate change can disrupt entire ecosystem structures through the disappearance of key species. Therefore, identifying environmental characteristics that limit species distributions is important for inferring potential responses to future environmental change [1,2]. This is particularly relevant for the management of species of conservation concern undergoing range shifts/contractions [2–4], or endemic species with naturally restricted ranges or limited dispersal abilities that will not be able to find new habitats (e.g. mountain species, flightless island species [5]). Moreover, it also applies to common species whose decline or redistribution may disrupt ecosystem structure, function and services [6].

Additionally, as species' habitat selection is scale-dependent, the spatial scale of investigation also influences our determination of how environmental factors shape species’ distributions [7–9]. For example, the presence of a species at the regional scale provides information of the long-term suitability of broad environmental parameters (e.g. temperature, rainfall, vegetation type), whereas local surveys can allow us to determine how fine-scale habitat features might influence specific population parameters (e.g. habitat complexity and suitability for reproduction; e.g. [10–13]).

The Tasmanian native hen (*Tribonyx mortierii*) is a unique species that is both a common keystone species and a flightless island species. It is an endemic rail of Tasmania, and one of the rare survivors of major prehistoric and historic extinction events that hit island and flightless birds worldwide [14,15]. The native hen is widespread in Tasmania and is one of the only two flightless rails to be at ‘Least Concern’ of extinction under the IUCN Red List [16]. However, this species suffered a complete extirpation from the Australian mainland 3500 years ago (suggested to be partly due to a great climatic variation along with predation by the newly arrived dingo and hunting by Aboriginal people [17]). It currently remains extant only on the large southern Australian island of Tasmania (approx. land area of 65 022 km^2^) and some of its offshore islands. In Tasmania, native hens exist across a range of environmental and climatic conditions, found from sea level up to 1100 m elevation [18]. Within this range, they are dependent on short-grass pastures (feeding almost exclusively on grass) and nearby freshwater to settle, survive and reproduce [18,19]. Moreover, being flightless and now endemic to Tasmania, the future dispersal potential of native hens is limited under future environmental shifts (e.g. climate change).

Climatic conditions in Tasmania are expected to intensify under climate change, including hotter and drier summers, altered rainfall regimes [20], and increased frequency of extreme climatic/weather events like droughts and heatwaves [21]. This is particularly relevant for native hens, with recent research suggesting that drought can cause severe decline in their population [22]. However, except for some local-scale ecological studies [18,19,23], little is known about the regional-scale environmental characteristics influencing the distribution of native hens in Tasmania and how expected changes in these may influence the distribution and survival of this island endemic rail. Despite their perceived ubiquity, the geographical range of native hens across Tasmania has not been explored in detail.

As climate models for Tasmania predict a shift towards a hotter, drier and more ephemeral rainfall environment over this century, we hypothesize that the distribution of native hens will contract, with populations disappearing from some currently occupied areas (being acute in areas suffering from water deficits). Here, we measure the importance of local factors on native hen population structure and breeding activity with surveys of three geographically separated local populations spanning a wide rainfall gradient. We then report the range of distribution records of native hens across Tasmania and undertake habitat suitability modelling to assess their distribution under contemporary climatic conditions, using land-use, climatic and topographic layers. Finally, we examine future predicted suitability using climate projections for 2055 and 2085 to evaluate to what degree the distribution of native hens in Tasmania would shift.

## 2. Methods

### 2.1. Local-scale factors measurements (fieldwork)

We selected geographically distant populations presenting different rainfall profiles during the late-autumn to spring period, April–November (electronic supplementary material, figure S1), as rainfall is an important factor for native hens' survival and reproduction [18,22]: ‘East’ (wukaluwikiwayna/Maria Island National Park; 42°34'51″ S 148°03'56″ E), ‘North’ (Narawntapu National Park; 41°08'53″ S 146°36'52″ E), and ‘West’ (adjacent to the town of Zeehan (712 inhabitants); 41°53'03″ S 145°19'56″ E). The period April–November corresponds to the six-month period preceding the middle point of the breeding season generally used for native hens' surveys (electronic supplementary material figure S1) [19,22].

All three populations were surveyed between 10 and 22 November 2019 (late spring, in the middle point of the breeding season) to determine population structure (total number of groups, group composition (number of adults and young), and breeding activity). Each population was monitored over 2 to 5 days, depending on habitat complexity and extent of the population area, until all native hens in the area had been surveyed, i.e. when the territories’ structure was found identical at least four times for populations with no previous data (North and West), and at least two times in well-known populations (East) [22], over two different half-days.

To align with methods used by Lévêque [22], we used territory mapping [24,25] as native hens maintain year-round territories and population sizes were measurable with our survey methodology. Territory mapping consists of establishing the location of birds over a number of visits to obtain distinct clusters representing each territory. Boundaries are determined by vocal disputes between neighbours, which are frequent in native hens. During each survey, a minimum of two observers conducted repeated group identification, based on location, neighbours' location and number of individuals per group (from two to five individuals per group in this study). The number of individuals and their age category (fledgling, juvenile or adult) were recorded per territory.

The total pasture area surveyed per population, and the total pasture area occupied by native hens were: North population: 2.0 km^2^ (1.3 km^2^ occupied); West population: 1.5 km^2^ (0.7 km^2^ occupied); East population: 1.5 km^2^ (0.6 km^2^ occupied). We measured environmental characteristics in the native hens’ territory following methods established by Goldizen *et al*. [19] to obtain quantitative measures of (i) protection cover, (ii) water availability, and (iii) food availability; these parameters are important for native hen reproduction [19].

— Protection cover was determined as the length (m) of the interface between dense patches of bushes and pasture, used by native hens for hiding and protecting chicks against predators [22]. It is an important parameter for breeding success [19]. We measured the total protection cover available to native hens in each population using satellite data from Google Maps (www.google.com/maps, accessed on 9 December 2019).
— For measures of food availability (grass) on territories, we selected random transects of a total length of 1 m across all territories (East: *n* = 15, North: *n* = 26, West: *n* = 22). Measurements of vegetation characteristics were measured and recorded every 2 cm along each transect, including the percentage of (i) total vegetation cover, (ii) green vegetation, (iii) vegetation cover that was grass, (iv) vegetation cover that was moss, and (v) the grass height (average length of grass blades). The same observer (L.L.) recorded all measures.
— Water availability on territories was recorded as territories that had access to water (running or stagnant) at the time the surveys were undertaken.
— Rainfall data were collected from the Bureau of Meteorology (BOM; www.bom.gov.au/climate/data) at the three population sites: North population at Port Sorell (Narawntapu National Park, 4 km away from the population site), West population at Zeehan (West Coast Pioneers Museum), East population at Maria Island (Darlington). Rainfall was reported as the amount of rainwater that had accumulated (i) during the six months prior to breeding season midpoint (31 October 2019) following Goldizen *et al*. [19] and (ii) during summer (December–February). Information on recent droughts (on a 3- to 11-month period prior to 31 October 2019) was assessed using values of rainfall percentile deficiency (below the 10th percentile) from BOM (http://www.bom.gov.au/climate/drought/#tabs=Rainfall-tracker). The 6-, 7- and 12-month periods were not accessible. BOM defines the category ‘serious deficiency’ as rainfall that ‘lies above the lowest five per cent of recorded rainfall but below the lowest ten per cent (decile range 1) for the period in question’, and ‘severe deficiency’ as ‘rainfall is among the lowest five per cent for the period in question’.

### 2.2. Species distribution modelling

#### 2.2.1. Data preparation

We collected presence-point data for native hens across Tasmania from the Atlas of Living Australia (ALA: www.ala.org.au; accessed 19 February 2021). We additionally included data from BirdLife Tasmania, the Department of Primary Industries, Water and Environment (DPIPWE) reports, and our personal observations, resulting in a total of 23 923 occurrences. Our study area included the Tasmanian mainland and nearby islands; however a large area from the southwest of Tasmania was removed where native hen distribution is not well documented. They are thought to be rare or absent in this region due to large proportion of button grass vegetation creating unsuitable habitat (electronic supplementary material, figure S2). All subsequent analyses were undertaken in Program R v4.0.4 [26].

Duplicates were removed by converting presence points into grid presences at 1 km^2^ resolution and retaining one native hen observation per grid (*n* = 2447 grid points after this step). Occurrences were visually inspected for any potential errors/outliers from outside Tasmania and Tasmanian islands: this removed seven false occurrences on King and Flinders islands and two observations in freshwater inland lakes (Lake Crescent and Great Lake).

As true-absence records were mostly unavailable, we generated pseudoabsences for sites where other land-bird species had been recorded (indicating observation effort at that point), but without native hen detections [27–29]. Native hens are large-bodied, ground-dwelling, active in the day and have a loud, distinct call, all of which accounts for a high detectability, if present at a location. We extracted these data from ALA, with 780 499 possible observations on the Tasmanian mainland and all nearby islands. We then excluded all grid cells with a native hen presence and removed any records within 3 km of native hen records: this value was chosen because it is the dispersal distance under which a native hen can naturally move outside of its territory [18]. This process resulted in 3222 pseudoabsence grid points.

Citizen-science datasets offer unique opportunities to study a species distribution using ‘crowd-sourced’ effort; however they tend to be access-biased and have non-random, clustered observations, leading to overrepresentation of certain regions and biases towards some environmental conditions (usually near urban areas [30]). One way to reduce spatial autocorrelation is to selectively de-cluster occurrences in biased areas using a pre-defined (minimum linear) nearest minimum-neighbour distance (*NMD*) [31]. As un-urbanized, sparsely populated areas have the least spatial point clustering (and hence spatial bias), the average number of observations in low human density areas provides the threshold number of records that can be used to tune and select the optimal *NMD* [28]. Therefore, we subdivided our data on a grid of 25 km^2^ cells to be relevant to the metric of human density and used the median of population density index (excluding cells < 1 human km^−2^) to define thresholds for low and high density. Population density was extracted from the ‘2011 Census of Population and Housing across Australia’ (bit.ly/3bth7W9). ‘Low density’ was defined as <6 people km^−2^ and ‘high density’ as ≥6 people km^−2^ (electronic supplementary material, figure S3).

There were 1111 presence points in areas of high human density and 1152 points in areas of low human density. We used the average number of native hen presences in low density (0.42/grid cell) as a threshold for (relatively) un-clustered observations. We calculated thinning distances for data in high- and low-density grids using the ‘thin’ function in R package spThin [32], and repeated randomly resampling runs to maximize the number of occurrences until the threshold value was achieved. Presence and pseudoabsence data were thinned separately. Using 20 re-sampling runs, 6 km was determined to be the optimum distance between high-density points and 4 km between low-density points. Following thinning using these distances, a total of 160 presence points in high-density cells and 517 in low-density cells were retained. For pseudoabsences, distances of 6 km in high-density cells and 5 km in low-density cells performed best for reaching approximate parity between the number of absence and presence points. The final total of presences fitted in the models was 677 and for pseudoabsences was 702 (electronic supplementary material, figure S4).

#### 2.2.2. Modelling parameters and variable selection

We selected 11 environmental raster layers (table 1) as predictor variables that may influence habitat suitability and native hen distribution in Tasmania. We normalized and centred all continuous variables using the 'normImage' function from the RStoolbox R package.

**Table 1.** Environmental predictors considered to develop species distribution models for the Tasmanian native hen (*Tribonyx mortierii*) in Tasmania. All raster layers were scaled at 1 km resolution. Asterisk (*): predictors retained after variable selection in the models.

predictor	description	data source
bioclimatic		
*mean diurnal temperature range	mean of monthly (max. temp.–min. temp.)	Biodiversity & Climate Change Virtual Laboratory (BCCVL; bccvl.org.au), summarized from 1976 to 2005
isothermality	(mean diurnal / temperature annual range) (×100)	
mean winter temperature	mean temperature during the wettest quarter of the year	
*mean summer temperature	mean temperature during the dry quarter of the year	
*precipitation seasonality	variation (standard deviation) in monthly rainfall over the year	
*summer precipitations	precipitations during the warmest quarter of the year	
topographic		
*elevation	elevation in metres above mean sea level	Department of Agricultural Resources (www.data.daff.gov.au)
*top roughness	terrain ruggedness, index of how variable the elevation is within a grid cell	created by Grant Williamson, University of Tasmania
landscape		
*land use	type of land/habitat modification	CLUM data (https://bit.ly/3GGWl3w), reclassified by Matthew Fielding (DEEP resources, University of Tasmania)
*land-use categories*	*conservation/reserves; modified native; farming; urban; plantation; mining and other human*	
*vegetation	extent and distribution of major vegetation types in Australia	NVIS data (https://bit.ly/2zguhry), reclassified by Matthew Fielding (DEEP resources, University of Tasmania)
*vegetation categories*	*rainforest; tall eucalyptus forest; woodland; shrubland; grassland; human/other*	
distance to freshwater	distance (Euclidean) of each raster cell from the nearest lake or river layer	created by Stefania Ondei (DEEP resources, University of Tasmania) using surface hydrology data (https://bit.ly/3x6A6Pd)

We undertook initial modelling and variable selection using random forest (RF) decision trees, as this machine-learning algorithm typically has good prediction accuracy in species-distribution contexts [33] and has been recommended as an efficient tool for variable selection [34–37]. We ran RF using the package caret, using repeated *k*-fold cross-validation (with *k* = 10 folds and 25 repetitions), and a suitability threshold of 0.5. We used the Pearson's correlation coefficient to analyse the correlation among predictor variables (with a cutoff at 0.7) using the 'removeCollinearity' function of the virtualspecies package. No intercorrelation between selected predictor variables was found. We selected eight variables resulting in the most accurate and parsimonious predictions using RF, maximizing AUC (area under the curve of receiver operating characteristic curve; table 1). ‘Distance to freshwater’, ‘mean winter temperature’, and ‘isothermality’ were the three variables (table 1) not included in the subsequent models.

We then used four statistical/machine-learning models to build the final species distribution models (SDMs): generalized linear model (GLM), generalized additive model (GAM), RF, and boosted regression trees (BRT). We used the caret package to tune each model parameters separately, using repeated *k*-fold cross-validation (with *k* = 10 folds and 25 repetitions), and a threshold of class probability of 0.5. We selected the best tuning parameters by maximizing out-of-sample AUC results (electronic supplementary material, table S1). We then used an unweighted ensemble model with the four models using the ensemble function from the package sdm.

### 2.3. Species distribution modelling for climate projections

As projections of future vegetation and land use were unavailable for Tasmania, we created a simpler model of current climate to project future climate (topographic and climate model) which excluded vegetation and land use. We retained all other variables, including elevation and top roughness as they were constant variables influencing distribution and are unlikely to drastically change in the future. We repeated the same tuning process with the new selection of predictor variables and the same tuning parameters were kept for boosted regression trees (electronic supplementary material, table S1). The *mtry* parameter (i.e. the number of variables randomly sampled as candidates at each split) changed for random forest (*mtry* = 1, a stub, also known as a ‘weak learner’). We used the projected environmental layers (bioclimatic and topographic) for the years 2055 and 2085 from two global climatic models (GCMs) ‘micro3_2_medres’ and ‘ukmo_hadgem_1’ (accessed from BCCVL; bccvl.org.au). These two models were recommended for their good model performance and independence [38]. All raster layers were scaled at 1 km^2^ resolution. We used the ‘topographic and climate only’ current model (model 2) to project future native hen distribution for two representative concentration pathways (RCPs): 8.5 (high CO_2_ emissions trajectory) and 4.5 (intermediate scenario), and for each, we ensembled the results from the GCMs (i.e. averaging all predictions of the fitted models into one model).

## 3. Results

### 3.1. Description of fine-scale predictors

The three native hen populations surveyed had different habitat quality, population structure and breeding activities (table 2). The East population had the most adults, with the West and North supporting lower (and similar) numbers (table 2). The West population had the highest proportion of juveniles, the greatest proportion of breeding groups and had six to eight times more rainfall than the two other populations (table 2). This was reflected by a more extensive vegetation cover and taller grass, as well as a higher quality of forage grass (%green grass; table 2). The lower grass cover in the West was due to the presence of moss in pastures (table 2). The North population was wetter than the East (in terms of both rainfall and % territories with water; table 2) and breeding activity was not detected at the time of the survey and appeared low in the East population (table 2). Accumulated territories' protective cover, an environmental feature associated with increasing survival of young [19], was highest for the West, which also had the most young hens, including some older juveniles, while it was the lowest for East, with few young birds, all very young chicks (table 2).

**Table 2.** Population structure, reproduction state, and environmental measures of territories of three native hen populations in Tasmania. See electronic supplementary material, figure S1, for population locations. North = 26 transects, West = 22, East = 15.

	populations		
	North	West	East
no. groups	11	12	17
no. groups > 2 adults	4/11	0/12	6/17
%breeding groups^a^	0	42	6
no. juveniles/no. adults	0/26	14/21	2/43
accumulated territories' bush edge (m)	11.04	14.38	8.66
grass height (cm)	1.87 ± 0.02	4.57 ± 0.07	1.58 ± 0.02
%vegetation cover	91.2 ± 2.4	98.5 ± 1.1	86.1 ± 3.8
%green vegetation cover	83.8 ± 1.7	99.9 ± 0.1	92.1 ± 1.7
%grass cover	94.2 ± 1.3	90.8 ± 2.1	97.0 ± 0.8
%moss cover	1.67 ± 0.99	16.9 ± 3.95	1.66 ± 0.62
%territories with water	73	100	53
rainfall (mm, April–Nov)	382 [600–900]^b^	2325 [1200–1800]^b^	297 [400–600]^b^
mean summer rainfall ± s.d. (mm, Dec–Feb 2006–2019)^c^	116 ± 50	351 ± 49	151 ± 79

^a^Groups with juveniles.

^b^Range for the period 1961–1990.

^c^Years missing: North: 2009; West: 2007 and 2016; East: 2006, 2007, 2008 and 2010.

#### 3.1.1. Droughts

The North and East populations had a ‘serious’ to ‘severe’ deficiency of rainfall over the 11 months before the surveys (electronic supplementary material, table S2). The North population experienced rainfall deficiency for all periods assessed: severe deficiency over the 3- and 10-month periods, and serious rainfall deficiency over the 4-, 5-, 8-, 9- and 11-month periods. The East site had serious deficiency on the 10- and 11-month periods only, while the West site had never been in rainfall deficit during the last 11 months (electronic supplementary material, table S2).

### 3.2. Broad-scale predictors: potential current distribution

**Figure 1.**
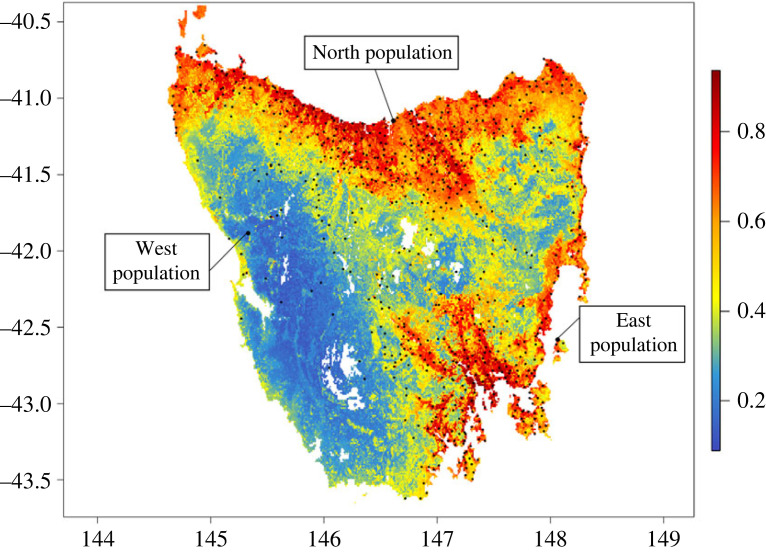
Habitat suitability map for native hens in Tasmania, produced by the ensemble species distribution model (SDM) projections on the current climate using bioclimatic, topographic and landscape predictor variables. The scale represents the habitat suitability index, ranging from 0: least-suitable habitat, to 1: most-suitable habitat. The black dots are the Tasmanian native hen occurrences used in the model. The arrows point to the three studied sites where field data on breeding and activity were collected.

Our SDM ensemble performed adequately when predicting the current habitat suitability for native hens across Tasmania (model-averaged AUC = 0.72, averaged TSS = 0.40, figure 1, electronic supplementary material, figure S5). About 37% of Tasmanian landmass (approx. 25 814 km^2^) was predicted to contain suitable habitat (greater than 50% suitability) for the species.

**Figure 2.**
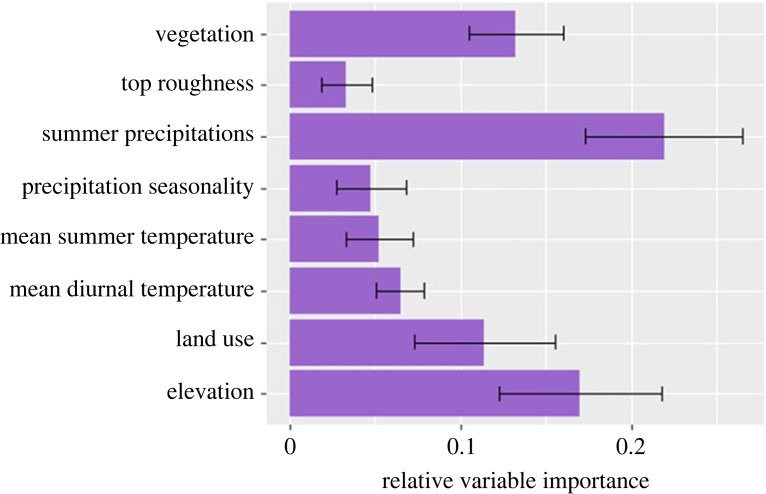
Variable importance (based on correlation metric) for the model average ensemble (GAM, GLM, RF, BRT) of the species distribution model (SDM) projections for the native hens on the current climate in Tasmania. Variables' importance was extracted from the getVarImp function of the R package sdm.

**Figure 3.**
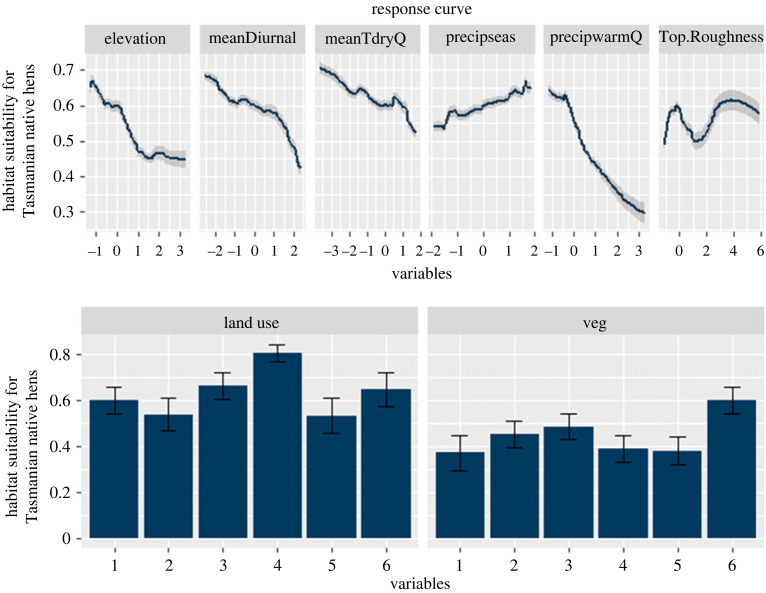
Response curves of the variables used in the species distribution model (SDM) projections on the current climate in Tasmania. Land use categories are: 1: Conservation/Reserves, 2: Modified Native, 3: Farming, 4: Urban, 5: Plantation, 6: Mining and Other Human. Vegetation categories are: 1: Rainforest, 2: Tall Eucalyptus Forest, 3: Woodland, 4: Shrubland, 5: Grassland, 6: Human/Other. All variables were normalized and centred. Variables' abbreviations: ‘meanDiurnal’: mean diurnal temperature range, ‘meanTdryQ’: mean temperature during the dry quarter of the year (summer), ‘precipseas’: precipitation seasonality, ‘precipwarmQ’: precipitations during the warmest quarter of the year (summer), ‘veg’: vegetation types.

Of the eight predictors, four climate and environmental layers were influential (greater than 0.1 relative variable importance, RVI) in the model (figure 2). Summer precipitation was the most important predictor of habitat suitability in the model (RVI 22%), followed by elevation (RVI 17%), vegetation type (RVI 13%) and land use (RVI 11%; figure 2). More specifically, native hens appear to prefer lower elevations and lower rainfall during summer and so the areas of high elevation and high rainfall, such as across south-west and central Tasmania, were less suitable (habitat suitability index less than 0.3; electronic supplementary material, figure S6). Native hens were more strongly associated with areas that have been heavily modified by humans (e.g. urban, farming, mining and other human types), compared to conservation reserves (figure 3) and thus were more prevalent around the main urban and agricultural areas in the south-east—especially around the capital Hobart—and along the north coast of Tasmania, around the two other largest northern cities (Launceston and Devonport; figure 1).

**Figure 4.**
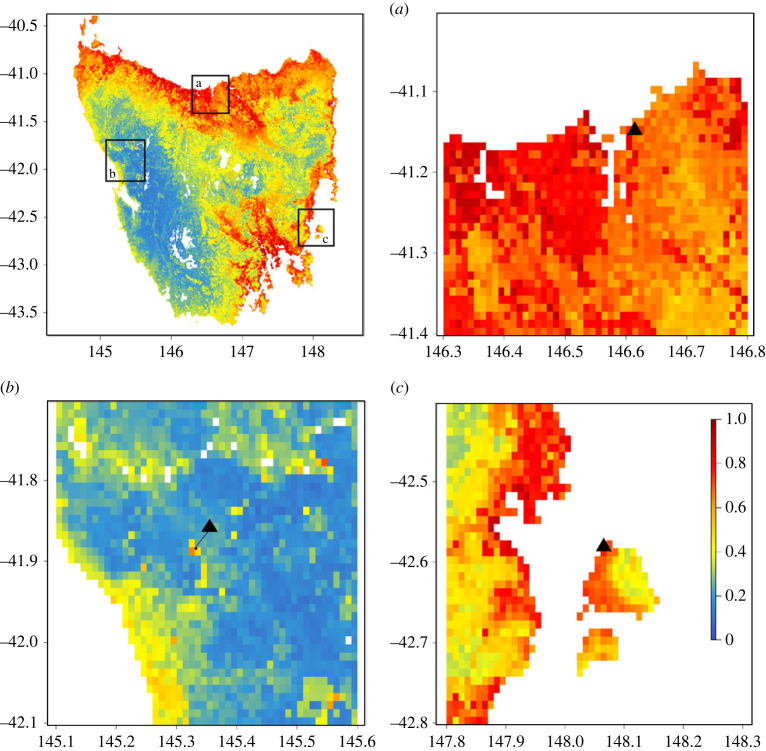
The location of the field sites we studied for Tasmanian native hen populations, situated on the habitat suitability map produced by the species distribution model using current climate and bioclimatic, topographic and landscape variables. The triangle symbols localize the populations: A, ‘North’ population; B, ‘West’ population; C, ‘East’ population.

We additionally evaluated how well our SDM matched with our observations from surveys at the three study sites. The West population was found within a pocket of medium suitability area (50–60% suitability) according to the SDM, surrounded by far less suitable habitat (figure 4). The North and East populations were located in high-suitability areas (greater than 70% suitability; figure 4).

### 3.3. Future climate projections

When removing landscape variables (vegetation and land use), the climate predictors were still reasonable approximations of the native hens' current distribution (electronic supplementary material, figures S7 and S8). This climate-only ensemble model (averaged AUC = 0.70, averaged TSS = 0.38; electronic supplementary material, figure S7) was used with bioclimatic and topographic predictor variables to model future projections. The climate in Tasmania is predicted to shift in various geographically specific ways (i.e. substantial regional variation in the extent of change) between now and the year 2055 (electronic supplementary material, figure S9). The north of Tasmania is predicted to have decreased seasonal precipitation and diurnal temperature but an increase in summer rainfall. In the centre-east of Tasmania, rainfall in summer will also increase as well as summer temperatures (electronic supplementary material, figure S9).

Regardless of the emission scenario (RCP 4.5 or RCP 8.5; electronic supplementary material, figure S10), areas of suitable habitat for the native hens are predicted to shift between now and the years 2055 and 2085. By 2055 (RCP 8.5), we found 37 674 km^2^ (54.3%) of Tasmania would gain suitability (greater than 0%), 6174 km^2^ (8.90%) would gain greater than 5% suitability and 396 km^2^ (0.57%) of Tasmania would gain greater than 10% suitability. The maximum suitability increase found was of 16.4%. On the other hand, 31 708 km^2^ (45.7%) would lose suitability (greater than 0%) by 2055, 7350 km^2^ (10.6%) would lose greater than 5% suitability and 2210 km^2^ (3.04%) would lose greater than 10% suitability. The maximum suitability decrease found was of 31.8%. Overall, under climate change alone, native hens are predicted to lose 1297 km^2^ of range at greater than 50% suitability and 330 km^2^ of range at greater than 70% suitability by 2055.

**Figure 5.**
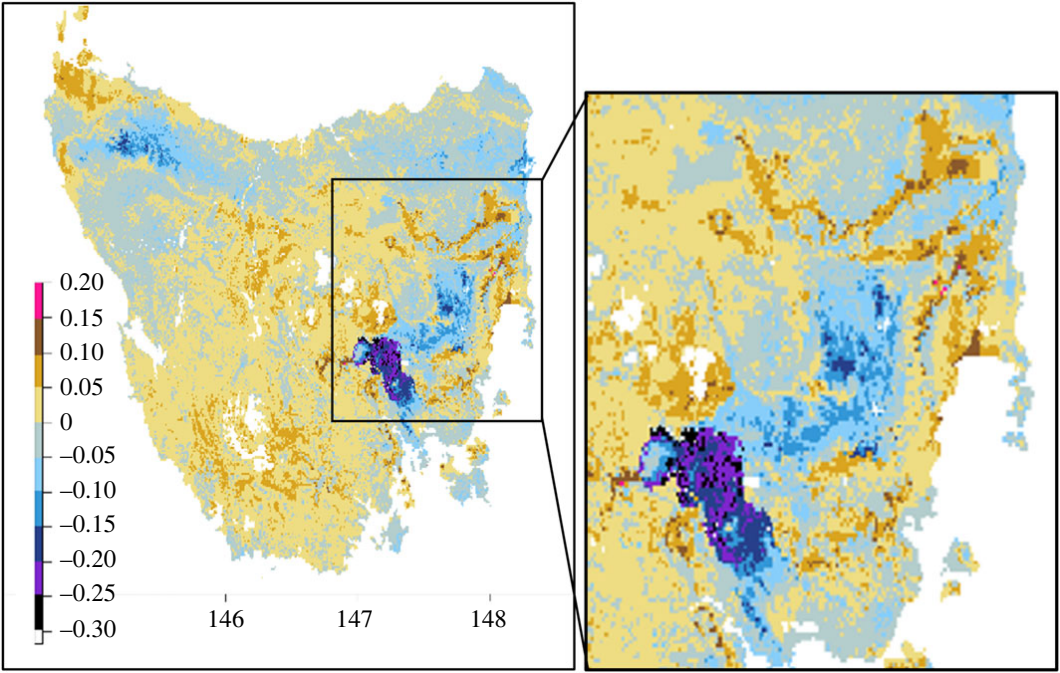
Predicted changes in environmental suitability for Tasmanian native hens between present projections and future predictions for the 8.5 RCP emissions scenario for the year 2055. The future suitability map predictions are averaged from the two GCMs ‘micro3_2_medres’ and ‘ukmo_hadgem_1’. The ‘warm’ colours indicate an increase in suitable habitat from present and ‘cool’ colours indicate a decrease in suitability.

Forecast shifts in native hen distribution are associated mainly with the shifting pattern of summer rainfall in some regions of Tasmania (electronic supplementary material, figure S9). Predicted changes in distribution and relative suitability were broadly similar for both years 2055 and 2085 (electronic supplementary material, figures S10 and S11). Parts of the north and east coast are predicted to decline in habitat suitability (with suitability decreasing by 5 to 15%). A localized pocket of suitability is predicted to decrease to a substantially greater extent in south-east Tasmania (area in purple, figure 5), following increases in temperature and precipitation in summer (electronic supplementary material, figure S9), as increases in these two variables corresponded to decreases in suitable habitats (figure 3).

## 4. Discussion

The Tasmanian native hen of southern Australia is a unique species that is both a common keystone species and a rare surviving flightless island species. Therefore, understanding the factors shaping its vulnerability is essential to mitigating further extinction events of endemic species or those with either restricted ranges or limited dispersal abilities. In this case study, we found that low precipitation in summer, low elevation, human-modified vegetation and proximity to urban areas were the key factors mediating the species’ current distribution. The three populations that we surveyed across the island state had distinct environmental characteristics with respect to habitat quality, population structure, and breeding activity. The population in the west, while located in the middle of a region of relatively low macroecological habitat suitability, nevertheless had the best habitat resources locally (longest bush edge, highest grass height and percentage of vegetation cover, green vegetation cover, and territories with water), and consequently, more breeding activity. Clearly then, fine-scale autecological information matters [9–11]. Across the species range, we found that future changes in temperature and precipitation are likely to lead to a decrease in area of 1297 km^2^ by 2055 from the current 25 814 km^2^ range at greater than 50% suitability (i.e. only 5% of the area of occupancy). This suggests that most populations of native hens might not shift their range much and are likely to be resilient to climate change. Collectively, this research reinforces the perception that native hens have benefitted from pastures created by European colonists of Tasmania [39], reinforces the idea that native hens are dependent on local water sources for success, and suggests that this flightless rail may be at a disadvantage from climate change in their endemic range in conditions where water stress increases.

The preferred habitat of the native hen includes areas with short-grass pastures and high availability of freshwater sources. Therefore, the distribution of farms and urban areas (generally composed of gardens, parks, golf courses, paddocks and with access to water) in Tasmania is likely to provide ideal habitats for native hens, while areas of high human impact (e.g. land clearings) are unlikely to be detrimental. Rainfall is an important factor for native hens' survival and reproduction [18,19], and Tasmania experiences wide variation in rainfall across the state (electronic supplementary material, figure S6). The wettest region of the island encompasses most of the Tasmanian Wilderness World Heritage Area (TWWHA), characterized by high-elevation mountains and distinctive vegetation types (temperate rainforests, sedgelands and herblands [40]), which together, constitute poor-quality habitat for the native hen. By contrast, across the east side (lowlands) of Tasmania (e.g. the midlands region), there is lower rainfall, many small towns, a dominance of open woodland and native grassland vegetation types, and extensive areas of land cleared for farming [41], all of which makes for good quality habitat for the native hen.

A key finding of this study is the importance of local water sources for native hens. While our broad-scale habitat suitability model matched the observed native hen distribution well, indicating wetter areas of the state are often less suitable for this species, field research at individual sites suggests that locally, the area with the highest summer rainfall (West) contained a native hen population that was the most demographically viable (in terms of breeding activity). By contrast, study sites with lower rainfall (the North and East populations) were located in areas predicted by the model to be highly suitable, yet measurements at the local scale revealed low to no breeding activity, suggesting that these populations were experiencing constrained growth due to dry conditions (see [22]). Interestingly, the variable ‘distance to water’ was not selected as important in our broad-scale models, reflecting native hens' capacity to rely on very small sources of water that can be as small as puddles or artificial drinking troughs (that are not currently captured at the resolution of our current spatial layers). Overall, it is clear that drought can be very influential for the viability of native hen populations, as these extreme precipitation events can cause the water sources to dry up, causing populations to decline over time [22]. This could explain why the North population, having experienced extended and consistent drought episodes over the last year, had no breeding activity detected. We do caution that there might be high variability across local sites, if more could be studied, and that our models were using climate averages, while rainfall anomalies were influencing the local surveys, illustrating that our broad-scale model does not always reflect the native hens’ habitat suitability. Overall, this research suggests that land use, and especially well-watered urban areas, has the potential to create ‘oases’ of suitable habitats for the native hens in a regional landscape of environmental constraints. This is possibly due to the native hens' capacity for high fecundity, allowing local populations to survive despite urban stressors [42].

Previous work on native hens has suggested the presence of bush edges adjacent to open pastures as being an important factor for the survival of young [19]. Indeed, observations from the West population, combining the longest length of bush edge and the highest number of young, supported the idea that urban land use and high rainfall, coupled with an abundance of vegetation cover (as a form of protective shelter), promotes breeding and recruitment. The East population had a few young, suggesting that while some territories were able to support breeding groups, either the overall poor conditions of the surrounding landscape have limited reproduction on other territories of the population, or that the chicks and young could have died before we did our survey due to the increased mortality stemming from a prolonged absence of water sources and/or the unobserved impact of predators [19]. Further research is needed to disentangle the mechanisms underpinning breeding variation in and across native hen populations.

Our SDM was adequate for predicting the geographical distribution of native hens across Tasmania. However, we note that this SDM also lacked some important information on the frequency of potentially important extreme events, notably droughts and heatwaves which can have deleterious impacts on birds [43,44]. Native hens are generalists, with an evolutionary potential to adapt to a range of climates (sub-fossil remains suggest that its historic distribution on mainland Australia during the Oligo-Miocene spanned from temperate south Australia to the tropics [17,45]). However, because of limited dispersal (since they are flightless) and strong territoriality, native hens might be especially vulnerable to local stresses and eventually local extinctions. Indeed, the partial return of dry climatic conditions to the Australian continent has been hypothesized to be an exacerbating factor facilitating extirpation of native hens from the Australian mainland [17], and this vulnerability is supported by recent observations [22]. Since native hens are strongly associated with specific climatic conditions, their future pattern of distribution is expected to shift following environmental changes such as summer rainfall. Based on climatic variables only, the habitat suitability for native hens is predicted to decrease overall across their Tasmanian range, most notably in areas that are presently highly suitable. Moreover, this will have the potential to be exacerbated by other factors such as the frequency and/or intensity of extreme events and the native hens’ low genetic diversity [46,47], which could limit their potential to withstand the deleterious effects of major environmental changes or adapt to them [48,49]. However, as human settlements and land use can buffer unsuitability over large regions, future distribution will also depend on how these develop in Tasmania.

Local and regional scales complement each other at different levels to better characterize habitat requirements [11]. A clear way to enhance our SDM would be through consideration of local-scale predictor variables, for example, local availability of water sources, pasture–bush interface habitats and local-scale native hen population density and reproduction. Such a ‘regionally suitable’ habitat model could illustrate long-term suitability for native hen presence and in doing so, indicate whether decolonization from surrounding areas is possible after, for instance, a local extirpation caused by an episode of extreme events (e.g. drought). In this context, future research might include hierarchical models, which although more complex to fit, offer the benefit of incorporating both regional and local scales [50–52]. However, to parameterize such a multi-level model, we would require surveys of native hen populations across a wide range of environmental and ecological conditions (e.g. predatory pressure, climate variation and intensity, inbreeding depression, etc.), to cover the full range of possible factors driving the species' demographics.

### 4.1. Future recommendations and conclusion

Tasmanian native hens are a rare case of a surviving flightless, island endemic rail species, which seem to maintain secure populations in Tasmania now and potentially into the future (by the years 2055 and 2085). Extreme climatic events and low genetic diversity could undermine their future resilience, but these are yet to be formally tested. Conversely, human land use (e.g. expansion of irrigated pastures) could help buffer these deleterious effects and promote suitable habitats. The potentially positive outlook this research presents for native hens should, however, be tempered by unpredictable external factors which we did not account for. For example, a malicious illegal introduction of the red fox (*Vulpes vulpes*) to Tasmania from the Australian mainland could be problematic, flip this equilibrium, and potentially threaten the Tasmanian native hen [47] in ways beyond the scope of this study. This could be analogous to how dingoes likely participated in the extinction of the native hens from the Australian mainland during the Holocene [17]. Nevertheless, native hens are a remarkable example of flightless rail species surviving anthropogenic activities, and even benefitting from living alongside humans.

## Data Availability

The data used in this article can be accessed from the Dryad Digital Repository: https://doi.org/10.5061/dryad.2jm63xsv8 [53]. The data are provided in electronic supplementary material [54].
